# Dietary Supplementation of Fermented Rice Bran Effectively Alleviates Dextran Sodium Sulfate-Induced Colitis in Mice

**DOI:** 10.3390/nu9070747

**Published:** 2017-07-13

**Authors:** Jahidul Islam, Takuya Koseki, Kouichi Watanabe, Ardiansyah, Slamet Budijanto, Akira Oikawa, Md Alauddin, Tomoko Goto, Hisahi Aso, Michio Komai, Hitoshi Shirakawa

**Affiliations:** 1 Laboratory of Nutrition, Graduate School of Agricultural Science, Tohoku University, Sendai 980-0845, Japan; mdalauddin1181@gmail.com (M.A.); tgoto@g-mail.tohoku-university.jp (T.G.); mkomai@m.tohoku.ac.jp (M.K.); 2 International Education and Research Center for Food Agricultural Immunology, Graduate School of Agricultural Science, Tohoku University, Sendai 980-0845, Japan; watakoh@tohoku.ac.jp (K.W.); asosan@tohoku.ac.jp (H.A.); 3 Faculty of Agriculture, Yamagata University, Tsuruoka 997-8555, Japan; tkoseki@tds1.tr.yamagata-u.ac.jp (T.K.); oikawa@tds1.tr.yamagata-u.ac.jp (A.O.); 4 Cellular Biology Laboratory, Graduate School of Agricultural Science, Tohoku University, Sendai 980-0845, Japan; 5 Department of Food Technology, Universitas Bakrie, Jakarta 12920, Indonesia; ardiansyah@bakrie.ac.id; 6 Faculty of Agricultural Engineering and Technology, Bogor Agricultural University, Bogor 16680, Indonesia; slamet.budijanto@gmail.com

**Keywords:** fermented rice bran, short chain fatty acids, tryptophan, tryptamine

## Abstract

Rice bran (RB) is a major by-product of rice polishing and a rich source of bioactive compounds. Here, we investigated the anti-colitis effect of diet supplementation with fermented rice bran (FRB) in a murine model of ulcerative colitis. FRB was prepared by dual fermentation of RB using fungi and lactic acid bacteria. Colitis was induced in C57Bl/6N male mice (*n* = 8/group) by dextran sodium sulfate (DSS). Body weight change, disease activity index (DAI), histopathology score, tissue myeloperoxidase (MPO) activity, cytokine and chemokine transcript levels, and the production of short-chain fatty acids (SCFAs) and mucin in the colonic tissue were monitored. Based on histopathology scores, DSS induced severe mucosal inflammation, with an increased loss of crypts, and inflammatory cell infiltration in the control and RB groups, but not in the FRB group. MPO activity, thiobarbituric acid-reactive substance levels, and pro-inflammatory cytokine transcript (*Tnf-α*, *Il-1β*, *Il-6*, and *Il-17*) levels were significantly higher in the control and RB groups than in the FRB group. Thus, dietary FRB attenuated intestinal inflammation owing to elevated SCFAs and tryptamine production, which might regulate tight junction barrier integrity and intestinal homeostasis. These results suggest that FRB could comprise an effective potential preventive agent for ulcerative colitis.

## 1. Introduction

Inflammatory bowel disease (IBD), a chronic and relapsing inflammation of the gastrointestinal tract, encompasses two types of intestinal inflammation: ulcerative colitis (UC) and Crohn’s disease [1]. UC is a complex and debilitating disorder that is characterized by the presence of discontinuous lesions in the rectal and colonic mucosa [2]. While the incidence rate of UC is high in Western countries, its occurrence in East Asian countries, such as Korea and Japan, has also increased in recent years on account of an increasing intake of a Westernized diet, characterized by high protein and fat content, as well as excessive sugar intake, with lower fiber consumption [3,4].

Although the etiology and pathogenesis of UC remain obscure, increasing evidence suggests that the dysregulation of mucosal immunological function is responsible for the initiation and propagation of this disease. UC is predominantly triggered by the epithelial invasion of intestinal microbiota due to the loss of epithelial layer integrity [5]. The resulting overproduction of pro-inflammatory cytokines, e.g., tumor necrosis factor α (TNF-α), interleukin 1β (IL-1β), and IL-6, induces colonic tissue damage and ulceration of the colon [6]. The efficacy of conventional treatments varies and the commonly used drugs have long-term side effects [7]. Thus, an effective preventive agent should be investigated, that would reduce inflammation for extended periods of time. Prebiotics are safe, indigestible food constituents, benefitting the host by selectively enhancing the growth of commensal microbiota in the gastrointestinal tract, that have been used for the treatment of IBD [8].

Rice bran (RB) is a rich source of bioactive components, e.g., dietary fiber, vitamins, free amino acids, and antioxidants, which have a potential to promote gastrointestinal health [9]. RB is presently available in most regions of the world as a by-product of rice polishing. The usage of RB for human consumption is limited, however, and RB is discarded or used as animal feed [10]. Such RB treatments as fermentation, enzymatic processing, or fractionalization have been employed to increase its quality or render it edible for humans, in the form of a dietary supplement. Processed RB or fermented rice bran (FRB) are foodstuffs that are more enriched in such ingredients as protein, fiber, and phenolic compounds, than the usual raw bran [11]. Therefore, it is extremely important to investigate the basic physiochemical and functional properties of FRB and to identify the mechanisms of action for its application as a nutraceutical.

Several studies have investigated the anti-colitis effects of processed RB in animal models, focusing on rice bran oil [12], brown rice fermented by *Aspergillus oryzae* [5,13], enzyme-treated rice fiber [8], and RB fermented by *Saccharomyces cerevisiae* and *Lactobacillus plantarum* [3]. Processed RB is enriched for different ingredients to varying degrees because of different fermentation processes and preparation methods. Among these enriched ingredients, an essential amino acid, tryptophan, is considered an effective candidate agent against UC [7,14]. In addition, in the gut, tryptophan metabolites of the microbiota, e.g., indole-3-aldehyde, kynurenine, indole-3-acetic acid, and tryptamine, can act as ligands for aryl hydrocarbon receptor (Ahr), a transcription factor that regulates *IL-22* gene expression, controls autoimmunity processes, and promotes rapid recovery from colitis [15].

FRB may also stimulate the microbial production of short-chain fatty acids (SCFAs), especially acetic acid (AA), propionic acid (PA), butyric acid (BA), and lactic acid (LA), which are strongly associated with the colonic health in human [16]. SCFAs are produced by the microbiota in the course of breaking down complex carbohydrates, such as fiber, and are a primary source of energy for the enteric epithelium [16]. The intestinal microbiota has a profound impact on the host immune system; dysbiosis of bacterial populations and suppression of SCFA production has been implicated in UC [17]. Increased SCFA levels promote colonic epithelial cell proliferation, stimulate mucin production, and epithelial cell integrity. In particular, BA has been shown to induce colonic regulatory T cells and limit the innate immune cell-driven inflammation; it also has the potential to eliminate mutated epithelial cells through the induction of apoptosis [17]. These activities contribute to the maintenance of colonic homeostasis by regulating the intestinal barrier integrity [18]. The intestinal barrier integrity is crucial for maintaining the beneficial relationship between the host and the intestinal microbes; as it not only constitutes a physical barrier, preventing the entry of invading microorganisms, but also provide a defense mechanism by sensing pathogenic microorganisms or their toxins [19].

Intercellular tight junction (TJ) complexes consist of multiple proteins, including occludin (OCLN) and claudin (CLDN), which mainly determine the intestinal barrier integrity [20]. TJs are located at the apical ends of the lateral membrane of the epithelial cells; the loss of TJ barrier integrity is associated with the initiation and development of UC [20,21]. Another characteristic of human UC is a pronounced depletion of mucin-producing goblet cells and the mucus layer, correlating with an increased microbiota-induced colonic inflammation and disease pathology [22].

Dextran sodium sulfate (DSS) is commonly used in rodent models to chemically induce the intestinal inflammation [15]. DSS administration leads to weight loss, bloody diarrhea, and immune cell infiltration, and to increased production of inflammatory mediators, similarly to human colitis [6].

The purpose of the present study was to investigate the effects of dietary FRB supplementation on UC pathology in a murine model of DSS-induced UC. We used a unique FRB, in which RB was fermented by both *Aspergillus kawachii* and *Lactobacillus* sp., notably enriched with free tryptophan and tryptamine. We hypothesized that FRB supplementation would mitigate the symptoms of colitis and its clinical severity by enhancing the indole derivatives and colonic SCFAs. To evaluate the protective effect of FRB, we also determined the serum pro-inflammatory cytokines and colonocyte pro-inflammatory gene expression, which include genes responsible for tight junction’s barrier integrity, mucin production, and inflammatory cell infiltration in the inflamed sites. Our results indicate a protective effect of dietary FRB in DSS-induced UC in the mouse model, linked to modulation of colonic SCFAs and pro-inflammatory gene expression.

## 2. Materials and Methods

### 2.1. Materials

The components of AIN-93M standard diet were purchased from Wako Pure Chemical Industries, Ltd. (Osaka, Japan) and Oriental Yeast Co., Ltd. (Tokyo, Japan). DSS (MW > 40 kDa) was purchased from Sigma-Aldrich (St. Louis, MO, USA). Serum TNF-α and IL-6 levels were determined by using commercially-available mouse enzyme-linked immunosorbent assay (ELISA) kits provided by Diaclone SAS (Besancon CEDEX, France) and R&D Inc. (Minneapolis, MN, USA), respectively. Unless otherwise specified, all chemicals and solvents used were of analytical grade. RB and FRB were kindly provided by Sunbran Company (Tendo, Japan). We used commercial microorganisms for FRB production. *A. kawachii* was obtained from Akita Konno (Akita, Japan), and *Lactobacillus brevis*, *Lactobacillus rhamnosus*, and *Enterococcus faecium* from Koei-science (Wako, Japan).

### 2.2. Preparation of FRB

FRB was prepared by dual fermentation using fungi and lactic acid bacteria. RB was initially steamed and cooled to about 30 °C, after which a spore solution of *A. kawachii* (10^6^ spores/g of rice bran) was mixed and incubated at 30 °C in a fermentation chamber for 44 h. The solid-state culture obtained was named rice bran koji. A mixture of rice bran koji and rice powder (2:1) was saccharified using a four-fold amount of water at 56 °C for 12 h, heated at 85 °C for 15 min, and then cooled to about 30 °C and the solution was inoculated with a mixture of lactic acid bacteria (*L. brevis*, *L. rhamnosus*, and *E. faecium*) at a concentration of 0.01% (*w*/*w*). The solution was then incubated at 37 °C overnight and then heated at 85 °C for 15 min to obtain the FRB. The FRB solution was then filtered, lyophilized, and kept at −30 °C until use. The major differences in RB and FRB micronutrient contents are described elsewhere [11]. Details of the experimental diets (10% (*w*/*w*) of RB and FRB) are given in Table 1.

### 2.3. Animals and Treatments

Animal Care Committee of Tohoku University approved the experimental plan of the present study. All experiments were conducted according to the guidelines issued by this committee, and in accordance with the Japanese governmental legislation (2005).

Male C57BL/6N mice (age: 10–12 weeks) were used in the study. They were littermates, and were maintained with free access to a commercial diet (F2, Funabashi Farm, Funabashi, Japan) prior to the study. The mice were housed in a pathogen-free mouse colony (temperature: 23 ± 3 °C; relative humidity: 55% ± 10%; light cycle: 12 h/d), with free access to food and drinking water. Mice were provided a control diet (AIN93M standard diet for rodents) or the control diet supplemented with 10% (*w*/*w*) of RB and FRB, starting 4 d before the initial DSS administration (*n* = 8/group). After 4 d, fresh mouse feces were collected to determine the tryptophan, tryptamine, and SCFAs levels. Acute colitis was then induced in all groups by supplementing the drinking water with 3% (*w*/*v*) DSS for 12 consecutive days. Body weight, food intake, water intake, stool consistency, and rectal bleeding were monitored daily. The mice were sacrificed on the following day and the severity of colitis examined. Disease activity index (DAI) was determined by combining the stool consistency and stool blood scores [23].

### 2.4. Determination of Myeloperoxidase (MPO) Activity and Thiobarbituric Acid-Reactive Substance (TBARS) Levels

MPO activity, an enzymatic marker generally used for the quantification of the extent of inflammatory cell infiltration in the colonic tissue, was determined according to the manufacturer’s instruction (Biovision, Milpitas, CA, USA). Briefly, colonic samples were thawed and homogenized on ice in 20 volumes of phosphate-buffered saline (PBS) supplemented with 0.1% NP40, and processed following the manufacturer’s recommendations. MPO activity was expressed as mU/mg of tissue. Lipid peroxidation was measured based on the formation of TBARS [24].

### 2.5. Histopathology

For histopathological analysis, the distal colon was fixed in a 10% formalin solution, incubated in 70% ethanol, at 4 °C, and embedded in paraffin; the fixed tissues were sectioned (4-μm thickness), stained with hematoxylin and eosin (H and E) [15], and examined by light microscopy at 100× magnification. The histological score was calculated depending on the severity of epithelial cell damage, crypt damage, and the degree of infiltration of inflammatory cells [15].

### 2.6. Tryptophan, Tryptamine, and SCFA Analyses

Tryptophan and tryptamine content in the feces and serum of RB and FRB group animals were determined by fluorescence high-performance liquid chromatography (HPLC) [25]. SCFA levels were quantified using a previously published protocol, with slight modification [26]. Briefly, fresh fecal samples (100 mg) were weighed and suspended in 2 mL of 10 mM NaOH solution containing crotonic acid (CA) internal standard (50 µL of 250 µg/mL), homogenized for 1 min using a homogenizer, and centrifuged for 20 min at 1500× *g* at 4 °C. Supernatants were collected and fat-soluble compounds were removed by chloroform extraction. Prior to analysis, the samples were further diluted (10×) in 20 mM NaH_2_PO_4_ (pH 2.7). The separations were performed under isocratic conditions (mobile phase: 20 mM NaH_2_PO_4_) on an Atlantis C_18_ column (4.6 × 50 mm, 5 μm, Waters, Milford, MA, USA) at 30 °C, and with a flow rate of 0.5 mL/min. The HPLC run time for each sample was 35 min, and the injection volume was 20 μL, with UV detection at 214 nm. The analytical curves were constructed by calculating the regression line, and the linearity was defined by the coefficient of correlation (*R*^2^). All analytical curves were linear within the studied range, with *R*^2^ > 0.99. The limits of detection for AA, BA, LA, PA, and CA were 31.25 μM, 62.50 μM, 15.62 μM, 156.0 μM, and 2.5 μM, respectively. The recovery percentages of AA, BA, LA, PA, and CA were 103.13%, 91.61%, 96.26%, 92.25%, and 99.10%, respectively, indicating that the extraction procedure and HPLC-UV detection method were sufficiently precise, accurate, and sensitive for the quantification.

### 2.7. RNA Extraction and mRNA Quantification

RNA was isolated from the colonic tissue using the Isogen reagent (NIPPON GENE, Tokyo, Japan), followed by RNA cleanup using the RNeasy mini kit (QIAGEN, Tokyo, Japan) with optional DNase I treatment according to the manufacturer’s instructions. RNA purity was determined spectrophotometrically by measuring the absorbance at 260 nm in relation to that at 280 nm. RNA concentration was adjusted to 1 μg/μL with RNase-free water; for cDNA synthesis, the samples were pre-incubated with oligo-dT primer and dNTP (GE Healthcare) at 65 °C for 5 min; next, SuperScript III Reverse Transcriptase and RNaseOUT RNase inhibitor (Invitrogen, Carlsbad, CA, USA) were added, and the incubation was continued at 50 °C for 60 min. An aliquot of synthesized cDNA was used as the template in quantitative polymerase chain reaction (PCR) using an Applied Biosystems 7300 Real-Time PCR System (Applied Biosystems, Foster City, CA, USA). The target cDNAs were amplified using gene-specific primers and SYBR Premix Ex Taq (Takara Bio, Otsu, Japan). The relative mRNA levels were normalized to the amount of eukaryotic translation elongation factor 1α1 mRNA (*Eef1a1*) [15]. The following primers were used: *Tnf-α*: 5′-GACGTGGAACTGGCAGAAGAG-3′ (forward) and 5′-TCTGGAAGCCCCCCATCT-3′ (reverse) [NM_013693.3]; *Il-6*: 5′-AGAGGAGACTTCACAGAGGATACC-3′ (forward) and 5′-AATCAGAATTGCCATTGCACAAC-3′ (reverse) [NM_001314054.1]; *Il-1β*: 5′-CTGTGTCTTTCCCGTGGACC-3′ (forward) and 5′-CAGCTCATATGGGTCCGACA-3′ (reverse) [NM_008361.4]; *Ccl2*: 5′-GTTGGCTCAGCCAGATGCA-3′ (forward) and 5′-AGCCTACTCATTGGGATCATCTTG-3′ (reverse) [NM_011333.3]; *Cxcl1*: 5′-TTGTGCGAAAAGAAGTGCAG-3′ (forward) and 5′-TACAAACACAGCCTCCCACA-3′ (reverse) [NM_008176.3]; *Cxcl2*: 5′-CCAACCACCAGGCTACAGG-3′ (forward) and 5′-GCGTCACACTCAAGCTCTG-3′ (reverse) [NM_009140.2]; *Cxcr3*: 5′-GCTGCTGTCCAGTGGGTTTT-3′ (forward) and 5′-AGTTGATGTTGAACAAGGCGC-3′ (reverse) [NM_009910.3]; *Ocln*: 5′-CTTCTGCTTCATCGCTTCC-3′ (forward) and 5′-CTTGCCCTTTCCTGCTTTC-3′ (reverse) [NM_008756.2]; *Cldn-1*: 5′-TGAGCCTAGAAAAGAGCC-3′ (forward) and 5′-GCCACTAATATCGCCAGACC-3′ (reverse) [NM_016674.4]; *Cldn-4*: 5′-CCTCTGGATGAACTGCGTGGTG-3′ (forward) and 5′-GTCGCGGATGACGTTGTGAG-3′ (reverse) [NM_009903.2]; *Muc2*: 5′-GCTGACGAGTGGTTGGTGAATG-3′ (forward) and 5′-GATGAGGTGGCAGACAGGAGAC-3′ (reverse) [NM_023566.3]; *Il-17*: 5′-CTCCAGAAGGCCCTCAGACTAC-3′ (forward) and 5′-GCT TTCCCTCCGCATTGACACAG-3′ (reverse) [NM_010552.3]; *Eef1a1*: 5′-GATGGCCCCAAATTCTTGAAG-3′ (forward) and 5′-GGACCATGTCAATGGCAG-3′ (reverse) [M81088].

### 2.8. Statistical Analysis

**Figure 1:**
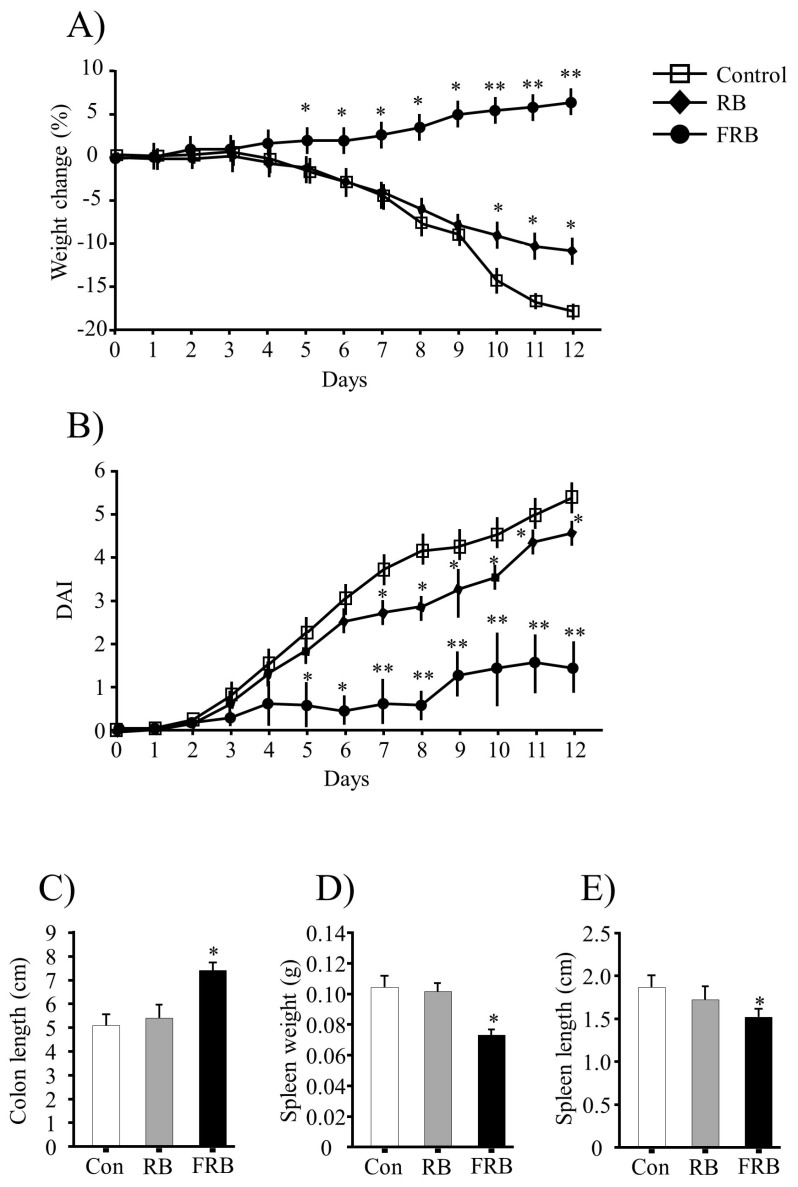
General evaluation of colitis. (**A**) Body weight change (%); (**B**) disease activity index (DAI); (**C**) colon length; (**D**) spleen weight; and (**E**) spleen length determination. Body weight loss was calculated as the percent difference between the original body weight (day 0) and the body weight on any particular day. The DAI was calculated based on the diarrheal score (0, normal; 1, mild soft; 2, very soft; and 3, watery stool) and the presence or absence of fecal blood (0, normal; 2, brown; 3, reddish; and 4, bloody stool). The values are expressed as the mean ± SEM (*n* = 8); one-way ANOVA was done followed by Dunnett’s test. * *p* < 0.05, ** *p* < 0.01, compared with the control (Con) group. RB, rice bran; FRB, fermented rice bran.

**Figure 2:**
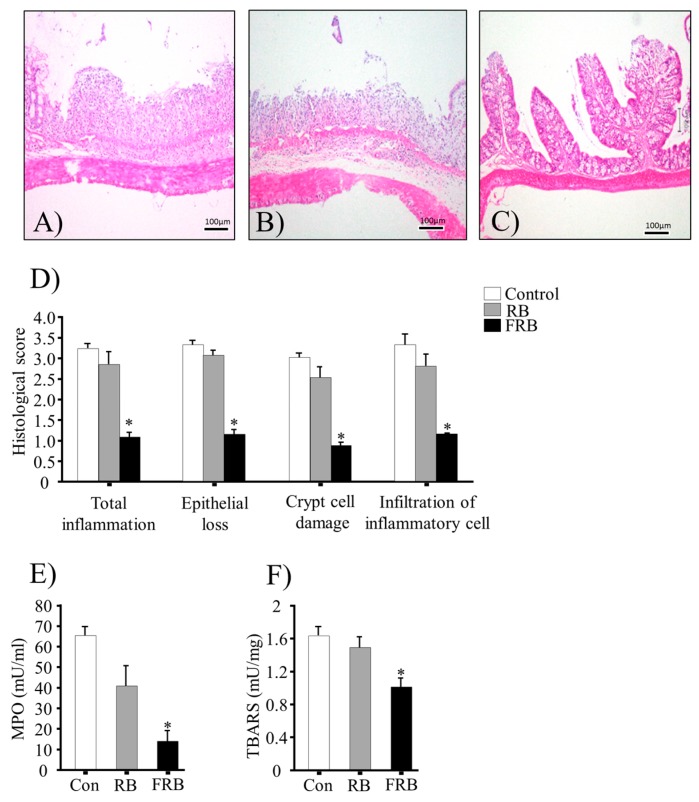
Dietary fermented rice bran (FRB) diet is associated with lower histopathology scores and inflammatory marker levels in dextran sodium sulfate (DSS)-induced colitis in the mouse model. Representative images (out of *n* = 4 in each group) of hematoxylin and eosin (H and E)-stained colonic tissues in (**A**) control; (**B**) rice bran (RB); and (**C**) FRB group animals; (**D**) histological scores. Histological scores were determined with following parameters: epithelial loss (0, none evident; 1, mild; 2, moderate; 3, severe; and 4, massive), crypt cell damage (0, none evident; 1, mild; 2, moderate; 3, severe; and 4, massive), and infiltration of the inflammatory cells in the mucosa (0, none evident; 1, mild; 2, moderate; 3, severe; and 4, massive). Scores were averaged to determine the total histological score in all parameters (five slides/sample); (**E**) Colonic myeloperoxidase (MPO) activity and (**F**) thiobarbituric acid-reactive substance (TBARS) values. The values are expressed as the mean ± SEM (*n* = 4); one-way ANOVA was done followed by Dunnett’s test. * *p* < 0.05, compared with the control (Con) group.

**Figure 3:**
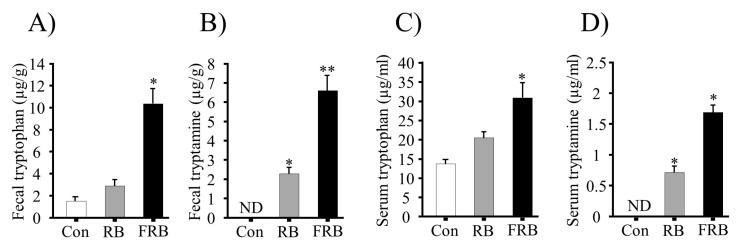
The involvement of fermented rice bran (FRB) in tryptophan and tryptamine production. (**A**) Fecal tryptophan; (**B**) fecal tryptamine; (**C**) serum tryptophan; and (**D**) serum tryptamine levels. The values are expressed as the mean ± SEM (*n* = 4); one-way ANOVA was done followed by Dunnett’s test. * *p* < 0.05, compared with the control (Con) group. RB, rice bran.

**Figure 4:**
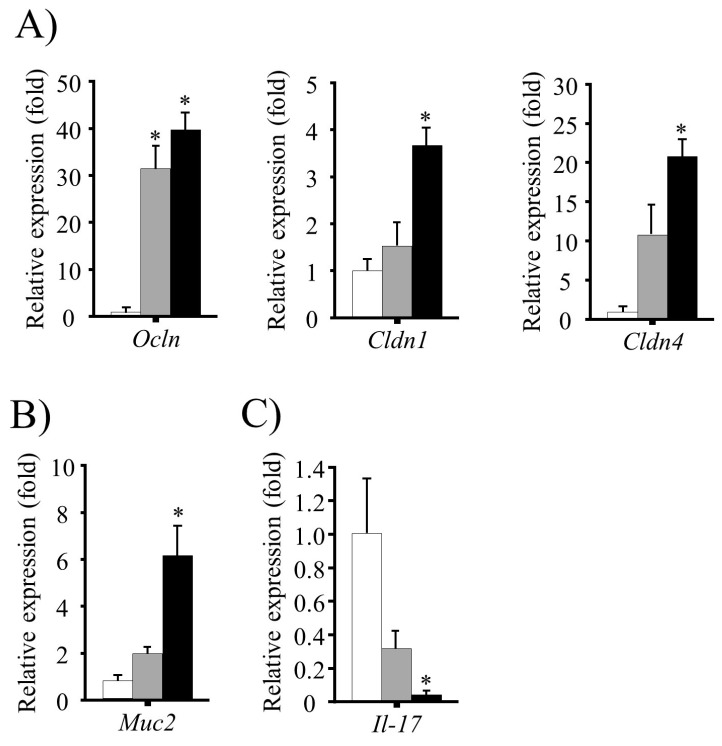
The role of fermented rice bran (FRB) in the intestinal barrier integrity. Colonic expression of genes encoding (**A**) tight junction proteins; (**B**) secretory mucin 2; and (**C**) *Il-17*. The mRNA levels were normalized to the expression of *Eef1a1* gene. The values are expressed as the mean ± SEM (*n* = 4); one-way ANOVA was done followed by Dunnett’s test. * *p* < 0.05, compared with the control (Con) group. RB, rice bran.

**Figure 5:**
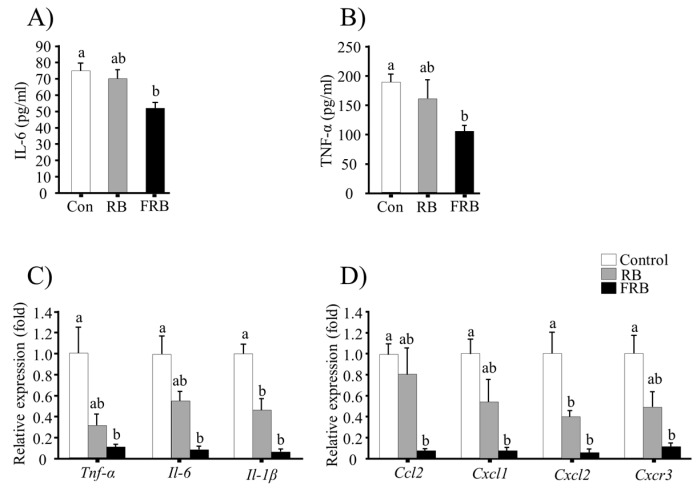
Evaluation of the anti-inflammatory effects of fermented rice bran (FRB). Serum levels of pro-inflammatory cytokines (**A**) TNF-α and (**B**) IL-6; (**C**) relative expression of colonic pro-inflammatory cytokine genes; and (**D**) relative expression of chemokine and chemokine receptor genes in the colon. The mRNA levels were normalized to the expression of *Eef1a1* gene. The values are expressed as the mean ± SEM (*n* = 4); One-way ANOVA was done followed by Tukey’s multiple comparison test. Different letters indicate significant difference. Con, control; RB, rice bran.

Numerical data are expressed as the mean ± standard error of the mean (SEM). Statistical evaluation was done by one-way ANOVA followed by Dunnet’s test (Table 2, Figure 1, Figure 2, Figure 3 and Figure 4) and Tukey multiple comparison test (Figure 5). The analysis was performed using SigmaPlot v. 12.5 (San Jose, CA, USA). A *p*-value < 0.05 was considered to indicate statistically significant differences.

## 3. Results

### 3.1. General Evaluation of Colitis

During the DSS treatment, significant body weight loss was observed in the animals from the control and RB groups but not in the FRB group. The percentage weight change in different groups is shown in Figure 1A. Following DSS challenge, the control and RB mice started to show clinical signs of illness earlier and the disease was more severe than in FRB mice. Watery stool and rectal bleeding were more severe in the control and RB groups (Figure 1B). Consistently with these observations, the DAI scores were higher in the RB group (*p* > 0.05, RB vs. control) than in the FRB group (*p* < 0.05, FRB vs. control) (Figure 1B).

After 12 d of DSS treatment, the mice were sacrificed and the colon lengths, and spleen lengths and weights were determined. The colon was significantly shorter in the control and RB groups (*p* > 0.05), than in the FRB group (*p* < 0.05 vs. control), as shown in Figure 1C. The spleen weights and lengths were significantly higher in the control and RB groups than in the FRB group (Figure 1D,E). Shortening of the colon in mice is correlated with histological changes, such as crypt cell damage, and colon length is often used as a morphological marker of the degree of inflammation [27].

### 3.2. Effect of FRB Diet on the Colonic Histological Score, MPO Activity, and TBARS Levels

Representative H and E-stained colon sections are shown in Figure 2A–C, and the histological scores (total inflammation, epithelial loss, crypt cell damage, and infiltration of inflammatory cells) are provided in Figure 2D. The results indicated acute inflammation in the colonic tissue of mice, with markedly higher histological scores in the control and RB group (*p* > 0.05, RB vs. control) than in the FRB group (*p* < 0.05, FRB vs. control). After the DSS challenge, MPO activity and TBARS values in the colon were higher in the control and RB groups than in the FRB group, as shown in Figure 2E,F.

### 3.3. FRB Diet Suppresses the Expression of Pro-Inflammatory Cytokines and Chemokines

Serum levels of the pro-inflammatory cytokines TNF-α and IL-6 were higher in the control and RB groups than in the FRB group (Figure 5A,B). The changes in *Tnf-α*, *Il-6*, and *Il-1β* mRNA levels in the colon are shown in Figure 5C; the expression of these pro-inflammatory cytokines was significantly higher in the control and RB groups (*p* > 0.05, RB vs. control) than in the FRB group (*p* < 0.05, FRB vs. control). Furthermore, FRB diet was associated with a significant reduction (*p* < 0.05, FRB vs. control) in mRNA levels of the chemokine genes *Ccl2* and *Cxcl1* and the chemokine receptor gene *Cxcr3* in comparison to those in the RB groups (*p* > 0.05, RB vs. control), as shown in Figure 5D. However, significant statistical differences were observed in *Cxcl2* mRNA levels in both RB and FRB groups compared to the control group (Figure 5D).

### 3.4. Effect of FRB Diet on Tryptophan, Tryptamine, and SCFA Production

Tryptophan levels were 5.5 mg/100 g and 31.3 mg/100 g, with tryptamine levels of 3.78 mg/100 g and 32.49 mg/100 g, in the RB and FRB, respectively. The high tryptophan and tryptamine content in the FRB supplementation in the diet increased the fecal tryptophan and tryptamine levels before DSS administration, as well as serum tryptophan and tryptamine levels after DSS administration (Figure 3A–D). Our analysis also revealed that the FRB diet increased the production of fecal SCFAs before DSS administration (Day 4), and colonic SCFAs after DSS administration (Day 12), as shown in Table 2.

### 3.5. Effect of FRB Diet on Intestinal Barrier Function and Mucin Production

The expression of genes encoding the TJ proteins OCLN, CLDN1 and 4, and the secretory mucin MUC2, were determined by reverse-transcription PCR. The mRNA levels of *Ocln* were higher in both FRB and RB groups (*p* < 0.05 vs. control), whereas *Cldn1* and *Cldn4* levels were higher in the FRB group only (*p* < 0.05 vs. control) as shown in Figure 4A. In addition, *Muc2* mRNA levels were found to be elevated in the FRB group (*p* < 0.05 vs. control), but not in the RB group (*p* > 0.05 vs. control) as shown in Figure 4B. In contrast, mRNA levels of the pro-inflammatory cytokine gene *Il-17* were lower in the FRB group than in the control and RB groups (Figure 4C).

## 4. Discussion

Dietary components have been recently revealed to exert remarkable beneficial effects on host physiology, and are currently targeted, or have high potential to be targeted, as treatments for human disease [28]. UC is a gastrointestinal disease with rapidly increasing world prevalence. It is important to identify the dietary compounds and its mechanisms to ameliorate the progression of inflammation during UC. In the current study, we demonstrated that FRB completely protects the animals against DSS-induced UC, by improving body weight and stool consistency, as well as decreasing intestinal bleeding, compared to the control and RB diets. Histological analysis revealed lower crypt damage and inflammatory cell infiltration in FRB group animals than in RB and control group animals.

Pro-inflammatory cytokines, such as IL-1β, IL-6, and TNF-α, amplify the inflammatory cascade of inflammatory mediators, destructive enzymes, and free radicals that cause tissue damage [29]. We observed elevated levels of TNF-α and IL-6 in the sera of RB and control group animals but not in FRB group animals. Furthermore, dietary FRB significantly inhibited the transcripts of these cytokines in the colonic tissue, similarly to another study [12].

Chemokines act as chemoattractants for neutrophils during acute inflammation and prolonged inflammation [30]. In the current study, we detected a significantly higher colonic expression of *Ccl2*, *Cxcl1*, *Cxcl2*, and *Cxcr3* genes in the control and RB groups (*p* < 0.05, RB vs. control) compared to the FRB group, which supports the protective effect of FRB in colitis.

MPO is an important bactericidal marker in UC; elevated MPO levels are responsible for greater neutrophil influx [15]. In the current study, MPO activity was significantly lower in the FRB group (*p* < 0.05 vs. control) than in the RB group (*p* > 0.05 vs. control). This indicated that the DSS-induced neutrophil infiltration in the colonic mucosa was suppressed by FRB supplementation.

The severity of chronic gut inflammation may result from a sustained overproduction of reactive oxygen metabolites and acute lipid peroxidation [31]. In the current study, we also observed a significant elevation of TBARS levels in the control and RB groups, which indicate severe tissue damage in the RB group (*p* > 0.05 vs. control), but not in the FRB group (*p* < 0.05 vs. control).

Although the fermentation process may enrich the protein and dietary fiber content in FRB [11], it is not known which specific FRB component contributes to the reduction of colitis. Here, during fermentation, FRB becomes enriched almost 10 times in tryptamine, which may act as an Ahr ligand [15]. Ahr has been highlighted as an immunological regulator in DSS-induced inflammation by promoting the differentiation of Th17, or regulatory T cells, and IL-22 production by Th22 T cells, which in turn regulate the epithelial barrier function and intestinal homeostasis [32]. The serum tryptophan levels were higher in the FRB group (*p* = 0.0101 vs. control), but not in the RB group (*p* > 0.05 vs. control). Low tryptophan levels are responsible for severe IBD complications and, thus, tryptophan containing diet supplementation comprises a novel therapeutic strategy for IBD treatment [7,14,15].

Although high levels of dietary fiber and prebiotics increase the intestinal SCFA concentration [33], few studies investigated the stimulation of SCFA production during UC by FRB. In the current study, fecal and colonic SCFA levels were significantly higher in the FRB group than in control and RB groups, as shown in Table 2. The differences in SCFA levels, especially BA and LA levels, clearly reflect the UC-ameliorating potency of FRB compared to RB. Generally, the absorption of SCFAs by the colonic cell is rapid, and the colon absorbs more than 95% of the SCFAs produced [34]. Cell-surface G-protein coupled receptors (GPRs), such as GPR41 and GPR43, are activated by SCFAs and exhibit anti-inflammatory effects to minimize inflammation. SCFAs, in particular BA, are known to induce apoptosis of T cells by inhibiting histone deacetylase, thus eliminating the source of inflammation in the colon [35,36]. The amount and relative abundance of SCFA in the colon may be considered as biomarkers of a healthy status [37]. Prebiotic substrates that selectively promote the growth of beneficial microbiota also induce changes in SCFA production in patients with irritable bowel syndrome [38]. Thus, FRB supplementation may alter the gut microbiota.

AA, PA, and BA enhance the mRNA levels of intestinal TJs genes, the major determinants of intestinal barrier integrity, and play an important role in intestinal defense, as well as in the maintenance of intestinal homeostasis [18,21]. Thus, the increased SCFAs levels by FRB supplementation may contribute to promote symbiotic intestinal environment, and prevent the development and progression of UC.

TJ proteins, especially OCLN, and CLDN1 and 4, are important for epithelial barrier integrity and seem to play a pivotal role in intestinal homeostasis [39]. Our data suggest that FRB supplementation significantly enhances the expression of genes encoding TJ proteins in the inflamed colonic tissue. Protection of the epithelial TJ barrier integrity by FRB involves the suppression of a chronic and robust activation of pro-inflammatory cytokines, such as TNF-α, IL-1β, and IL-6. We also observed that the expression of a gene encoding another pro-inflammatory cytokine, *IL-17*, which is responsible for augmenting autoantibody production in UC [40], was lower in the FRB group than in the RB and control groups. It is tempting to speculate that the modulation of epithelial-immune interactions by dietary FRB supplementation might present a novel preventive strategy for UC.

The mucus layer protects the gastrointestinal mucosa from mechanical, chemical, and microbial challenge [41]. Mucin 2 (encoded by *Muc2*) is the most prominent mucin secreted by the goblet cells [41]. High *Muc2* mRNA expression was observed only in the FRB group. Thus, in the control and RB groups, reduced *Muc2* expression in the intestine leads to abnormal tissue morphology, marked by an increased thickness of the gut mucosa, increased inflammatory cell infiltration, and robust pathogenesis of UC.

**Figure 6:**
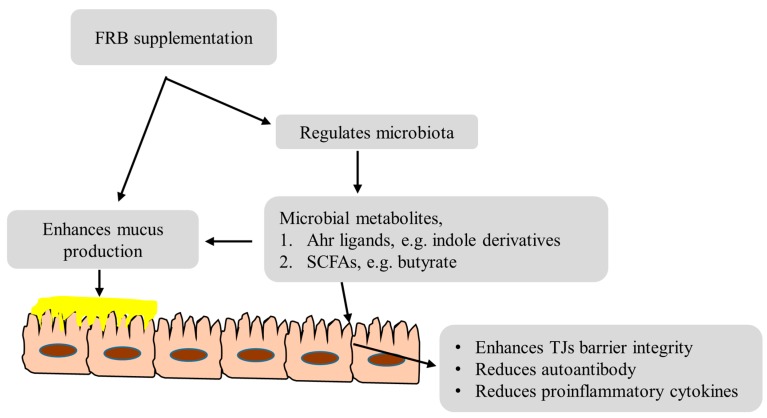
Prominent examples of FRB contributing to protect from DSS-induced colitis.

RB fermentation by *A. kawachii* increases the flavonoid content in FRB [42]; flavonoids are not only responsible for the smell and flavor, but also regulate innate immunity, inhibit the production of pro-inflammatory cytokines and, thus, reduce the severity of experimental colitis [43]. An overview of the mechanisms of action of FRB underlying the protection against DSS-induced colitis is shown in Figure 6.

In the present study, the effect of whole RB on UC is unclear. RB is rich in bioactive components, such as ferulic acid [12]. Ferulic acid has both antioxidant and anti-inflammatory properties and its anti-colitis effect in a DSS model has been reported [12]. Here, we have analyzed TBARS levels and it was found to be elevated in the RB group compared to that in the FRB group (Figure 2F). Thus, a dual fermentation process may augment the antioxidant properties in FRB.

In the future, more evidence on different FRB fractions and active components should be collected to clarify their mechanism of action. Metabolomics analysis might also reveal multiple potential protective mechanisms of FRB, including immunomodulation to prevent UC. We anticipate that FRB will emerge as an excellent source of nutraceuticals, as well as a functional food.

## 5. Conclusions

Dual fermentation of RB enriches its functional value. The nutritional benefit of RB fermentation is widely recognized, although the details remain unclear. A promising food component, FRB can be consumed by a large number of people, with health-promoting effects. The current study demonstrates the potential of FRB consumption as a dietary supplement for preventing intestinal inflammatory disorders.

## Figures and Tables

**Table 1:** Diet composition (g/1000 g).

Ingredient	Control Group	RB Group	FRB Group
*tert*-Butylhydroquinone	0.008	0.0072	0.0072
l-Cystine	1.8	1.62	1.62
Choline bitartrate	2.5	2.25	2.25
Vitamin mixture	10	9	9
Mineral mixture	35	31.5	31.5
Soybean oil	40	36	36
Cellulose	50	45	45
Sucrose	100	90	90
Casein	140	126	126
Cornstarch	620.70	558.6228	558.6228
Rice bran	–	100	–
Fermented rice bran	–	–	100
Total	1000.00	1000.00	1000.00

FRB, fermented rice bran; RB, rice bran.

**Table 2:** Concentration of short-chain fatty acids (SCFAs; μmol/g) in the feces and colon tissue.

	Feces			Colon		
	Control	RB	FRB	Control	RB	FRB
Lactic acid	1.65 ± 0.40	5.09 ± 0.86	6.11 ± 0.81 *	ND	ND	0.77 ± 0.05 *
Acetic acid	1.49 ± 0.39	3.71 ± 0.54	5.43 ± 1.38	2.24 ± 0.30	2.23 ± 0.16	3.64 ± 0.22
Butyric acid	ND	0.34 ± 0.15	0.87 ± 0.13 *	ND	ND	0.55 ± 0.02 *
Propionic acid	0.81 ± 0.22	2.67 ± 0.30	9.75 ± 1.50 *	3.80 ± 0.25	4.63 ± 0.37	5.69 ± 0.12 *

ND, not detected; FRB, fermented rice bran; RB, rice bran. Values are expressed as the mean ± SEM (*n* = 4); * *p* < 0.05 vs. control.
